# Divergent Evolutionary Profile of *MULE* Transposons in Arthropods

**DOI:** 10.3390/ani16132011

**Published:** 2026-07-01

**Authors:** Hong Chen, Shasha Shi, Kuilin Xiang, Quan Wang, Naisu Yang, Bo Gao, Chengyi Song

**Affiliations:** College of Animal Science and Technology, Yangzhou University, Yangzhou 225009, China; mx120230878@stu.yzu.edu.cn (H.C.); dx120220158@stu.yzu.edu.cn (S.S.); dx120250214@stu.yzu.edu.cn (K.X.); mx120230886@stu.yzu.edu.cn (Q.W.); naisu.yang@yzu.edu.cn (N.Y.); bgao@yzu.edu.cn (B.G.)

**Keywords:** *MULE*, transposon, arthropods, genome, evolution

## Abstract

*Mutator*-like elements (*MULEs*) are a major DNA transposon superfamily. The evolutionary profiles of *MULE* transposons and their impact on arthropod genomes remain largely unexplored. Here, we define the evolutionary landscape—including distribution, diversity, structure, and activity—of *MULE* transposons across 4268 assembled arthropod genomes. Systematic analysis reveals a divergent evolutionary profile. From the sampled genomes, 222 arthropod species harbor *MULE* transposons, comprising 322 distinct elements. Phylogenetic analysis divides arthropod *MULE* transposons into twelve clades. The majority of species (164) carry only one type of *MULE* transposon, while 51 species contain two or three types, and a few host multiple types (4–9). Copy numbers vary significantly, ranging from 5 to 88 per species. Structural variation is pronounced: full-length *MULEs* range from 1.4 kb to 10.0 kb, with most falling between 2.5 kb and 5.0 kb. Low Kimura divergence in several species suggests recent *MULE* activity, noteworthy because most transposons are expected to be inactive. Collectively, our results show that *MULEs* are widely distributed yet phylogenetically structured across arthropods, with lineage-specific expansions and recent activity in several species.

## Simple Summary

The evolutionary profiles of *MULE* transposons in arthropod genomes remain largely unknown. Analyzing 4268 genomes, we defined their distribution, diversity, structure, and activity. In total, 222 species harbor 322 distinct complete *MULEs*, whose transposases form twelve clades. Copy numbers range from 5 to 88, and lengths vary from 1.4 to 10.0 kb (mostly 2.5–5.0 kb). Kimura divergence suggests activity in several species. Our findings reveal the evolutionary dynamics of *MULEs*, providing a foundation for functional studies and applications.

## 1. Introduction

Arthropods represent a highly diverse and successful invertebrate phylum [1,2]. Transposable elements (TEs) are mobile DNA sequences that can move within genomes. They drive arthropod evolution by inducing mutations, causing chromosomal rearrangements, and facilitating horizontal gene transfer. The phylogenetic distribution of TEs often mirrors that of their hosts, reflecting long-term host–TE associations [3,4,5]. Arthropods have evolved defense systems, such as the piRNA pathway, which silences transposable elements at both the transcriptional and post-transcriptional levels and also provides antiviral immunity [6]. Significantly, this knowledge has been harnessed for genetic engineering and pest control using tools such as the *piggyBac* transposon [7]. Understanding these elements is essential for elucidating the dynamic evolutionary interplay between TEs and their host genomes.

*Mutator*-like elements (*MULEs*) are a superfamily of cut-and-paste DNA transposons, including *Mutator* and *Rehavkus* [8,9]. The *Mutator* system of TEs was first identified in maize, where it represents a highly mutagenic family of transposons; lines carrying large numbers of these elements can exhibit mutation frequencies 50 to 100 times the background level [10]. Because they transpose at high rates and target genic regions, these transposons can rapidly generate large numbers of new mutants, which has made the *Mutator* system a favored tool for both forward and reverse mutagenesis in maize [9]. The diversity, insertion specificity, and association with conjugation machineries of the *Mutator* superfamily in prokaryotes have been well defined [11]. *MULE* diversity across eukaryotic species has been characterized [12], and *MULEs* in plants—such as maize [13,14,15], *Medicago truncatula* [16], *Arabidopsis thaliana* [17], grasses [18], rice [19], barley [20], and sugarcane [21]—have been extensively reported. However, only a few reports are available for *MULEs* in animals. Evolutionary profiles of *MULEs* in the mosquito *Aedes aegypti* [22] and across 163 eukaryotic worm species [23] have been reported, but their evolution and impact on arthropod genomes remain systematically unexplored.

In this study, we systematically surveyed *MULE* transposons across 4268 arthropod genomes, analyzing their structural features, distribution patterns, and evolutionary classifications. Our results show that 222 species harbor complete MULE transposons, comprising 322 distinct elements, with several species showing signs of recent activity—a notable finding given that most TEs accumulate mutations and become inactive over evolutionary time. These findings reveal the diversity of *MULE* transposons in arthropod genomes and provide a foundation for future functional studies.

## 2. Materials and Methods

### 2.1. Retrieval of MULE Transposons

We downloaded 4268 assembled reference genomes of arthropods from the National Center for Biotechnology Information (NCBI) database (https://www.ncbi.nlm.nih.gov/, accessed on 5 December 2024). Using confirmed 478 *MULE* transposase core catalytic domain (DDE) representative sequences (FASTA file, Supplementary File S1) collected (clustered with 80% identity) from the literature and the RepBase [24] database (https://www.girinst.org/repbase, accessed on 15 December 2024) from eukaryotes as references, we performed homology searches against the constructed arthropod whole-genome assembly database using a local tBLASTN [25] (E-value ≤ 1 × 10^−20^, coverage ≥ 80%) to identify homologous *MULE* transposons. For the initially obtained DNA sequences, we extended 5 kb of flanking sequence on both sides. These sequences were clustered using USEARCH software (v11.0.667) with an identity threshold of 0.8 [26]. Clustered sequences were subsequently aligned at their boundaries using the MAFFT plugin implemented in BioEdit (v7.2.6) [27]. *MULE* types were defined based on sequence identity (≥80%) and structural features, specifically conserved terminal inverted repeats (TIRs) and target site duplications (TSDs), and copy numbers for each type were subsequently determined based on the alignment of each type of *MULE* in each species. Copies with complete flanking regions and a putatively intact transposase open reading frame (>300 amino acids) were classified as complete. This threshold is consistent with the typical architecture of *MULE* transposases, which comprise a ~180-amino-acid DDE domain and an N-terminal DNA-binding domain [11], and most eukaryotic homologs exceed 300 aa, as previously reported [12,28]. Based on these clustering results, we selected copies possessing terminal inverted repeats (TIRs) and a complete *MULE* transposase coding sequence as representative *MULE* sequences for the corresponding species, providing foundational data for subsequent molecular structure and phylogenetic analyses.

Sequences with low homology and low coverage were filtered out and excluded from the analysis. Although this conservative screening strategy improved result accuracy, it may have overlooked a substantial number of *MULE* transposon-related sequences that have undergone truncation, degeneration, or “domestication” by the host genome, including *MULE*-derived sequences lacking TIR features. Therefore, the actual diversity of *MULE* transposons in arthropods may be higher than currently predicted.

### 2.2. Phylogenetic Analysis

To investigate the molecular evolutionary relationships and family classification of the initially identified *MULE* transposons, we collected *MULE* transposase reference sequences from the literature and integrated them with the *MULE* transposase sequences (>300 amino acids) obtained above, plus *Rehavkus* sequences as an outgroup for rooting. Multiple sequence alignment was performed using the G-INS-I algorithm in MAFFT (v7.471) [29]. Subsequently, a maximum likelihood phylogenetic tree was constructed with IQ-TREE (v1.6.1) using an ultrafast bootstrap approach with 1000 replicates, with *Rehavkus* transposase as the outgroup [30]. The resulting tree file was uploaded to the iTOL [31] website (https://itol.embl.de/upload.cgi, accessed on 5 December 2024) for annotation and visualization.

### 2.3. Sequence Analysis

The MAFFT program was used to align sequences and determine transposon boundaries. Sequence alignment was performed using the ClustalW program in BioEdit software (v7.8.0) [27], and TIRs were manually determined. For *MULE* transposons with >5 copies, the ClustalW program in BioEdit (v7.8.0) was used to align copies from each species; after removing gaps, consensus sequences were constructed using DAMBE (v4.0.36) software [32]. The open reading frames (ORFs) of *MULE* transposons were predicted using BioEdit (v7.8.0) and the Genscan web server (http://hollywood.mit.edu/GENSCAN.html, accessed on 5 December 2024). Complete transposons with intact TIRs and a complete transposase (>300 amino acids) were used for subsequent functional structure determination and multiple sequence analysis. Predicted structural diagrams of each *MULE* transposase were drawn using IBS (Illustrator for Biological Sequences, v1.0.3) [33]. Multiple sequence alignments of DDE domains were performed using MAFFT, provided in Supplementary File S2, and results were visualized using BioEdit (v7.8.0) [27].

### 2.4. Evolutionary Dynamics Analysis

Genome annotation of consensus or representative *MULE* transposon sequences was performed using RepeatMasker to assess evolutionary dynamics. Kimura divergence (K) was calculated using the calcDivergenceFromAlign.pl script from the RepeatMasker package [34]. Data visualization was performed using GraphPad Prism (v8.0.2). This analysis reflects transposon activity on a relative timescale within each genome; younger transposons generally exhibit lower Kimura (K) divergence.

## 3. Results

### 3.1. Distribution of MULE in Arthropoda

Using the tBLASTn method described above, with the DDE sequences of known *MULE* transposons as references, this study systematically investigated the distribution of *MULE* transposons among 4268 arthropod species. Complete *MULE* transposons were identified in 222 species across 24 orders. Lepidoptera (88 species), Coleoptera (52 species), Hemiptera (17 species), Diptera (14 species), Orthoptera (5 species), Hymenoptera (3 species), Blattodea (2 species), Plecoptera (2 species), Trichoptera (2 species), Ixodida (3 species), and Decapoda (4 species) exhibited relatively broad distributions, whereas other orders contained fewer species harboring *MULE* transposons (Table 1 and Supplementary Table S1).

Based on the identification of 8498 mined *MULE* transposons, we identified a total of 322 *MULE* transposon types across 222 species. *Galeruca laticollis* and *Kuschelorhynchus macadamiae* each contained nine types of *MULE* transposons, representing the two species with the highest diversity. Additionally, *Eupithecia pulchellata*, *Apomyelois bistriatella*, *Rhagonycha fulva*, *Pammene aurita*, and *Cerapteryx graminis* harbored six, five, four, four, and four types of MULE transposons, respectively, indicating high *MULE* transposon diversity in these species. Only one type of *MULE* transposon was detected in 164 species, whereas two or three types were detected in another 51 species; these *MULE* transposon types possess distinct TIRs and transposases (Table 1 and Supplementary Table S1).

The copy numbers of *MULE* transposons varied significantly among species, ranging from 5 to 88 copies, with complete copies defined as those containing TIRs on both flanks and encoding a transposase of ≥300 amino acids. Among all 322 identified *MULE* transposon types, 305 contained intact TIRs and encoded a complete transposase, whereas the remaining 17 types, although possessing intact TIRs, encoded truncated transposases of only 236–295 amino acids in length (Table 1 and Supplementary Table S1).

### 3.2. Classification and Structure Organization of MULE Tn

**Figure 1:**
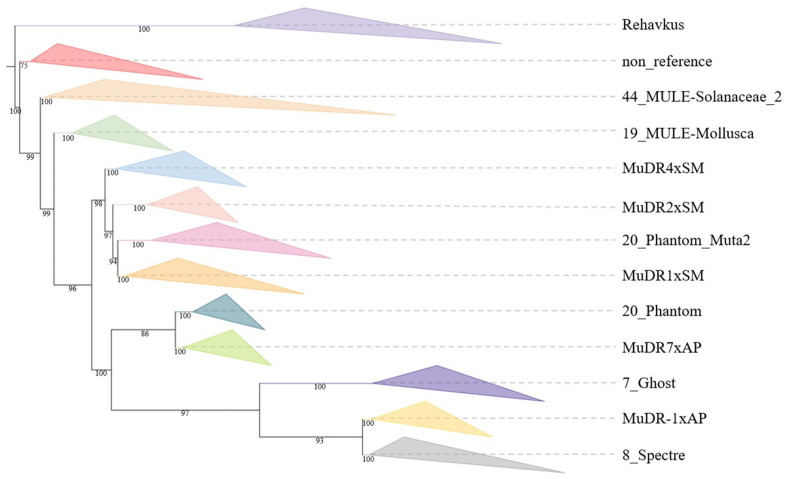
Evolutionary relationships of *MULE* transposon families. Phylogenetic tree of *MULE* families based on full-length transposase sequences. The tree was constructed using the maximum likelihood method implemented in IQ-TREE with ultrafast bootstrap support (1000 replicates). Rehavkus was used as an outgroup.

Based on the methods described above, a phylogenetic tree was constructed using the IQ-tree program, with known *MULE* transposase sequences from eukaryotes as references and the *Rehavkus* transposase as an outgroup for rooting. The results showed that *MULE* transposases could be divided into twelve clades (MuDR-1xAP, 8_Spectre, 7_Ghost, MuDR7xAP, 20_Phantom, MuDR1xSM, 20_Phantom_Muta2, MuDR2xSM, MuDR4xSM, 19_MULE-Mollusca, 44_MULE-Solanaceae_2, and non_reference), each with high bootstrap support (Figure 1 and Supplementary Figure S1).

The structural characteristics of *MULE* transposons from different species showed marked variation. The full-length of *MULE* transposons ranged from 1.4 kb to 10.0 kb, with the vast majority falling within the 2.5 kb to 5.0 kb range. Some species contained relatively short *MULE* transposons; for example, in *Lithobius variegatus* (Order *Lithobiomorpha)*, *Prosopocoilus inquinatus (Coleoptera)*, *Chrysomela saliceti* (*Coleoptera*)*, Cantharis nigra* (*Coleoptera*)*, Tipula vernalis* (*Diptera*), *and Phaonia angelicae* (*Diptera*), most *MULE* transposons were only about 1.8 kb to 2.0 kb in length. In several other species, such as *Apolygus lucorum* (Hemiptera), *Stegodyphus lineatus* (Araneae), *Gibbaranea gibbosa* (Araneae), *Crambus pascuella* (Lepidoptera), and *Eudemis profundana* (Lepidoptera), the full length of *MULE* transposons tended to be longer, mostly ranging from 5.5 kb to 7.0 kb, with the longest transposon reaching 9.9 kb (Table 1 and Supplementary Table S1).

**Figure 2:**
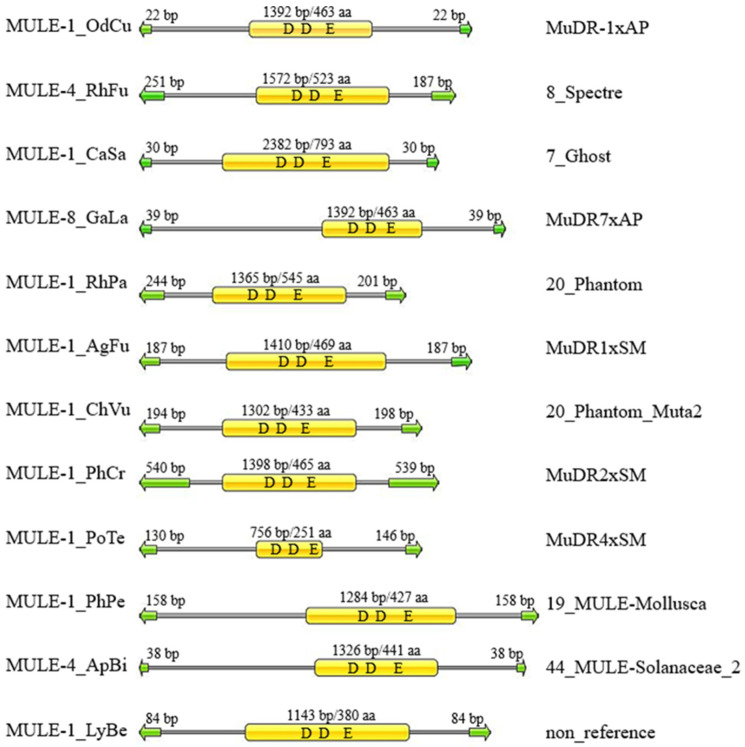
The domain architecture of *MULE* transposases is conserved across all families, featuring the characteristic RNase H-like catalytic domain (DDE) as defined in previous studies. Structural diagrams of representative transposons from the twelve *MULE* families.

Complete *MULE* transposons are flanked by terminal inverted repeats (TIRs) and encode a transposase of over 300 amino acids in the middle (Figure 2). The detected complete *MULE* transposons ranged from 1875 bp to 9972 bp in length, encoding a single open reading frame (ORF) of 313 aa to 1136 aa. In six species—*Longitarsus dorsalis*, *Dysauxes ancilla*, *Listronotus bonariensis*, *Photinus pyralis*, *Ceraclea dissimilis*, and *Anaspis frontalis*—two open reading frames (ORFs) were present, encoding a total transposase length ranging from 495 aa to 721 aa. Truncated transposons encoding transposases of only 236 aa to 295 aa were detected in species such as *Spicauda simplicius*, *Maniola jurtina*, *Muschampia baetica*, *Ochlodes sylvanus*, *Thymelicus lineola*, and *Erynnis tages*. In species including *Eretmocerus hayati*, *Eupithecia pulchellata*, *Eremobia ochroleuca*, *Polia nebulosa*, *Cosmia pyralina*, and *Cerapteryx graminis*, most of the detected transposons were complete copies, with transposase lengths ranging from 313 aa to 1136 aa (Figure 2 and Supplementary Table S1).

Among the *MULE* transposons identified in this study, different transposons exhibited significant variation in the length of terminal inverted repeats (TIRs), ranging from 13 bp to 1193 bp. Most of the longer TIRs were found in species such as *Caradrina kadenii*, *Aphodius fimetarius*, *Adelphocoris suturalis*, *Palaemon carinicauda*, *Lithobius variegatus*, and *Chrysomela saliceti*, reaching lengths of hundreds to thousands of base pairs, with the longest TIR being 1193 bp. In most species, TIR lengths typically ranged from 20 bp to 95 bp (Figure 2 and Supplementary Table S1).

*MULE* transposases mainly contain a catalytic domain (DDE) and a zinc finger domain (Zn-finger). As the core functional region of *MULE* transposases, the catalytic domain contains a highly conserved acidic amino acid triad, DDE (Figure 2 and Supplementary Figure S2). By analyzing all *MULE* transposase sequences selected from each family, it was found that among the twelve *MULE* families, except for the 7_Ghost family, in which the first and second aspartic acid residues are separated by 84 amino acids and gaps, the separation distance between the first and second aspartic acid residues in the remaining families generally ranges from 60 to 76 amino acids and gaps. Regarding the distance between the second aspartic acid residue and the third glutamic acid residue, the MuDR1xSM and MuDR4xSM families exhibit separations of 156 and 151 amino acids and gaps, respectively; the 20_Phantom_Muta2 family shows a separation of 172 amino acids and gaps; and the non_reference family displays a separation of 214 amino acids and gaps. For the other eight families, the separation distance between the second aspartic acid residue and the third glutamic acid residue generally ranges from 120 to 135 amino acids and gaps.

### 3.3. Mining Putatively Active MULE Transposons in Arthropods

Kimura divergence provides a relative timescale for transposon activity based on accumulated mutations [35]. Actively transposing or recently active elements exhibit low K-divergence, whereas ancient, inactive copies show high divergence. In this study, to identify young and highly active transposons, we selected *MULE* transposons with complete copy numbers (>5 copies possessing TIRs and an ORF > 300 amino acids), constructed consensus sequences as references, and used RepeatMasker to analyze Kimura divergence across multiple species. Our data mining revealed divergent evolutionary profiles of *MULE* transposons in arthropods.

**Figure 3:**
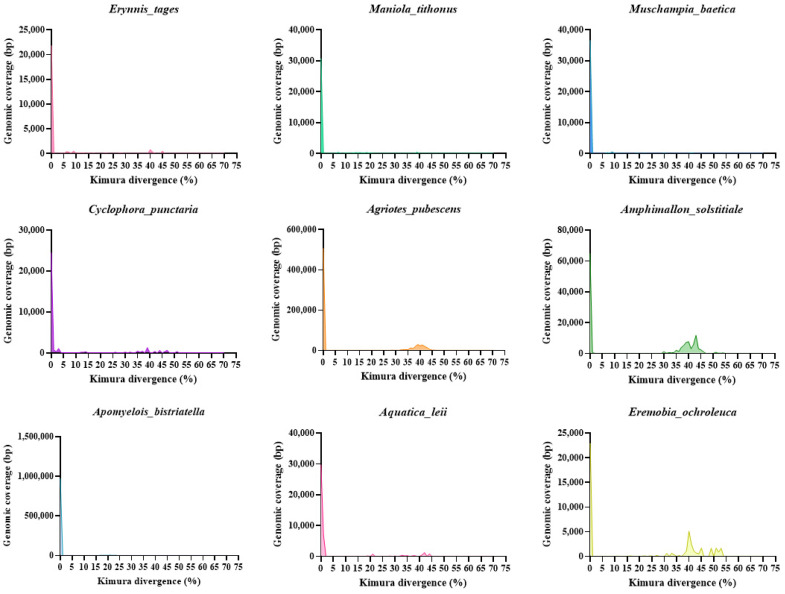
Evolutionary dynamics of *MULE* in arthropods. The Kimura divergence (K-value) was calculated using the RepeatMasker program based on consensus or representative sequences. The *y*-axis represents the coverage (in base pairs, bp) of each *MULE* transposon in the genome, and the *x*-axis represents the estimated Kimura divergence. Species names are indicated at the top of each panel.

Kimura divergence indicated that *MULE* transposons may be active in several species: MULE-1_MaTi in *Maniola tithonus*, MULE-1_ErTa in *Erynnis tages*, MULE-1_CyPu in *Cyclophora punctaria*, MULE-1_TiVe in *Tipula vernalis*, MULE-3_RhFu and MULE-4_RhFu in *Rhagonycha fulva*, MULE-1_LyCr and MULE-2_LyCr in *Lygephila craccae*, MULE-1_GrEq in *Grypus equiseti*, MULE-1_AgPu in *Agriotes pubescens*, and MULE-1_MyPa in *Mythimna pallens*. These transposons appear to have invaded genomes recently and may still retain transposition activity. Some *MULE* transposons showed evidence of long-term, repeated invasion over evolutionary time, including MULE-1_AgPu in *Agriotes pubescens*, MULE-1_CoCi and MULE-2_CoCi in *Corythucha ciliata*, and MULE-1_DoPl in *Dolomedes plantarius*. Additionally, multiple invasion events were observed for MULE-1_CoPy in *Cosmia pyralina*, MULE-1_CoCi in *Corythucha ciliata*, MULE-1_StBi in *Stictocephala bisonia*, and MULE-1_TeSi, MULE-2_TeSi, and MULE-3_TeSi in *Tenodera sinensis* (Figure 3 and Supplementary Figure S1).

## 4. Discussion

Mining *MULE* transposons via tBLASTn searches across 4268 arthropod genomes revealed that their distribution is phylogenetically non-random, showing clear clade-specific patterning. *MULEs* were predominantly found in *Lepidoptera* (88 species) and *Coleoptera* (52 species), accounting for 63% of all *MULE*-containing species. Species harboring multiple MULE types (e.g., *Galeruca laticollis* and *Kuschelorhynchus macadamiae*) are not closely related, suggesting that *MULE* diversity reflects lineage-specific ecological or genomic factors rather than host relatedness. Recently invaded species (low Kimura divergence) span multiple orders (*Lepidoptera*, *Diptera*, and *Coleoptera*) without clear phylogenetic clustering, implying that horizontal transfer or host defense differences may outweigh host phylogeny in shaping recent invasion patterns. Similarly, species with longer *MULEs* (>7 kb) or higher copy numbers (>50 copies) are distributed across multiple orders without obvious phylogenetic clustering. Collectively, *MULEs* are phylogenetically structured at the level of presence/absence, but their diversity, activity, and structural variation are largely lineage-specific and not simply explained by host evolutionary relationships.

Based on our structural analysis, the full length of *MULE* transposons in arthropods spans from 1.4 kb to nearly 10 kb. This substantial size variation not only reflects the high degree of differentiation within the *MULE* transposon family. Additionally, the observed size expansion in certain *MULEs* may be associated with their capacity to capture host gene sequences; however, other mechanisms may also contribute to this structural diversity. For example, transposons found in certain species of Hemiptera and Lepidoptera reached lengths of 7–9.9 kb, and their internal topological structures may be more complex than those of conventional transposons. Among all *MULE* transposons identified, TIR lengths showed great diversity, ranging from 13 bp to 1193 bp. Longer TIR sequences may provide multiple cooperative binding sites for transposases, thereby precisely anchoring integration positions in complex genomic environments and enhancing transposase binding efficiency. We observed that the first three base pairs at the TIR termini of most MULE transposons contain highly conserved G/C bases, suggesting that this feature may be related to recognition by *MULE* transposases. The species-specific differences in TIR length provide new perspectives for studying the coevolution of transposons and host genomes, and the underlying molecular mechanisms await further investigation.

At the catalytic core level, although transposases from each branch retained the typical DDE catalytic triad motif, the conservation of transposase domains varied significantly among different families, and protein folding patterns also differed. Furthermore, the widespread presence of zinc finger domains in most *MULE* transposons provides structural support for their DNA-binding stability and transcriptional regulation during transposition. This complex domain architecture—comprising catalytic, insertion, and zinc finger domains—collectively facilitates the evolution and horizontal transfer of *MULE* transposons in arthropods and also provides a theoretical basis for their development as novel large-cargo vector systems.

According to the transposon life cycle model, newly invading elements undergo copy expansion, followed by inactivation and degradation via mutation accumulation and negative selection, leaving few intact copies. Kimura divergence [35] tracks this mutation accumulation through base-substitution rates, serving as a reliable indicator of insertion age and a predictor of activity—supported by cellular assays where low divergence correlates with retained activity in transposons like *ZB* [36], *PS* [37], *Buster* [38], and *Spy* [39]. Recent work has also identified diverse candidate active MULEs in worms, with one native *MULE* from Aporrectodea caliginosa validated by a dual donor-helper assay [23]. Our Kimura divergence analysis revealed contrasting evolutionary patterns across arthropods. *MULEs* in *Agriotes pubescens* show stable, long-term occupancy, suggesting host–transposon equilibrium, while multiple activity peaks in Tenodera sinensis imply repeated invasions, possibly via horizontal transfer. Strikingly, species including *Maniola tithonus*, *Erynnis tages*, *Cyclophora punctaria*, *Tipula vernalis*, *Rhagonycha fulva*, *Lygephila craccae*, *Grypus equiseti*, *Agriotes pubescens*, and *Mythimna pallens* (Figure 3) harbor *MULEs* with extremely low divergence—very young copies that are likely active. Notably, these arthropod *MULEs* mirror recently invading worm *MULEs* [23] in both low divergence and intact structure (transposase and TIRs). Given that a worm *MULE* (*A. caliginosa*) actively transposes in human cells, our findings strongly suggest that these newly identified arthropod *MULEs* may be active as well. Future experimental validation—including RNA-seq to confirm transposase expression and functional assays to assess transposition in human cells—would greatly strengthen this inference.

Owing to their high transpositional activity and propensity to insert into or near genes, *MULEs* can act as potent mutagens, generating novel genetic variation upon which natural selection may act. Their ability to capture host gene fragments further facilitates exon shuffling and the emergence of new gene functions [9]. Our Kimura divergence analyses of *MULEs* in arthropods reveal that while some copies are ancient and have already shaped historical genome evolution, the widespread occurrence of low-divergence copies across multiple species points to ongoing transpositional activity. Thus, *MULEs* continue to drive arthropod genome evolution through insertional mutagenesis, chromosomal rearrangements, and gene capture, potentially fostering lineage-specific adaptations and overall genomic diversification.

## 5. Conclusions

Overall, the evolutionary profile (distribution, diversity, structure, and activity) of *MULE* transposons across 4268 arthropod genomes was defined. Potentially active *MULE* transposons may be present in multiple species, including *Galeruca laticollis*, *Kuschelorhynchus macadamiae*, *Eupithecia pulchellata*, *Apomyelois bistriatella*, *Rhagonycha fulva*, *Pammene aurita*, and others.

## Figures and Tables

**Table 1:** Taxonomic distribution of DDE/*MULE*.

Distribution	Number of Species Containing a *MULE*	Number of Species Containing an FL *MULE*	Number of Species Containing a Complete *MULE*	Length of the Complete MULE (bp)	Tpase Length of Complete *MULE* (aa)	TIR Length of Complete *MULE* (bp)
Total	222	218	202	1875–9972	313–1136	13–1193
*Lepidoptera*	88	84	75	2698–9206	313–518	24–1193
*Coleoptera*	52	52	47	1931–7070	321–903	14–1103
*Hemiptera*	17	17	16	2017–9078	366–834	30–1527
*Diptera*	14	14	13	2026–5280	361–487	72–431
*Orthoptera*	5	5	5	5050–5402	554–782	43–143
*Hymenoptera*	3	3	3	2172–4113	334–475	126–514
*Blattodea*	2	2	2	4093–7021	342–788	80–640
*Plecoptera*	2	2	2	3860–4041	510–589	76–103
*Trichoptera*	2	2	2	6085–6584	356–781	113–175
*Ephemeroptera*	1	1	1	6362	543	34
*Mantodea*	1	1	1	5755–6316	711–803	111–128
*Zygentoma*	1	1	1	4939–5256	366–456	101–249
*Ixodida*	3	3	3	2734–4214	339–500	87–144
*Mesostigmata*	1	1	1	4315	427	158
*Opiliones*	1	1	1	6214	742	391–405
*Decapoda*	4	4	4	2364–7555	437–1136	22–777
*Isopoda*	1	1	1	4297	462	192
*Stomatopoda*	1	1	1	3514	466	115
*Geophilomorpha*	1	1	1	7207	1061	136–141
*Lithobiomorpha*	1	1	1	1875–2320	471–491	106–222
*Poduromorpha*	1	1	1	3010	475	147
*Symphypleona*	1	1	1	6638	445	66
*Anostraca*	1	1	1	4672	455	92–112
*Siphonostomatoida*	1	1	1	4482	496	43

FL *MULE*: transposons flanked by detectable TIRs. TIR: terminal inverted repeat.

## Data Availability

The original contributions presented in this study are included in the article/Supplementary Materials. Further inquiries can be directed to the corresponding author.

## Supplementary Materials

The following supporting information can be downloaded at: https://www.mdpi.com/article/10.3390/ani16132011/s1, Figure S1: Evolutionary dynamics of *MULE* transposons in arthropods. The transposon insertion ages were estimated based on K divergences as described in methods. Temporal dynamics are visualized as K divergence (X-axis) versus genomic coverage (Y-axis). Different species are represented by distinct colors. Transposons more likely to be active are highlighted in red. (As a supplement to the main text (Figure 3), all transposons shown have a copy number greater than 5. (For details regarding the sequences associated with the figure, please refer to Supplementary Table S1); Figure S2: Alignment of amino acid sequences of partial *MULE* transposases from different clades in arthropods. The catalytic domain (DDE) is indicated. Representative sequences from each clade are shown (randomly selected). See Supplementary Table S1 for the complete set of sequences; Table S1: Statistical results of all MULE transposons in arthropods. The columns ‘Family’, ‘class’, and ‘order’ indicate the taxonomic classification of the host species and the superfamily categorization of the MULE transposon. The ‘species’ column denotes the specific arthropod species from which the transposon was identified. The ‘Name’ column provides the assigned full nomenclature for each element, consisting of the species name followed by a sequence number (e.g., Species_name-1), while ‘MULE-name’ gives the abbreviated name (e.g., MULE-1_SpAb). The ‘GCA_number’ specifies the NCBI genome assembly accession used for the search. ‘Copy number’ represents the total number of transposon copies identified within the genome. ‘Seq length (bp)’ and ‘seq identity %’ indicate the length of the transposon sequence and its internal sequence similarity, respectively. The ‘ORF length (bp)’ and ‘protein length (aa)’ columns detail the length of the predicted Open Reading Frame (nucleotide) and the translated transposase (amino acid) sequence. ‘TIR length (bp)’ and ‘TIR identity %’ describe the length and sequence conservation of the Terminal Inverted Repeats. The ‘Accession Number’ lists the specific NCBI accession ID for the retrieved transposon sequence; Supplementary File S1: Representative sequences of the MULE transposase core catalytic domain (DDE); Supplementary File S2: Multiple sequence alignment of MULE transposon DDE domains.
