# Supplementary material for: Divergent Evolutionary Profile of MULE Transposons in Arthropods
Source: Animals (Basel). 2026 Jul 1;16(13):2011. doi: 10.3390/ani16132011 (PMC13359780; doi:10.3390/ani16132011)
Supplement: Supplementary file 1 [file animals-16-02011-s001.zip › Supplement Materials.pdf]

SUPPLEMENTARY MATERIAL

Evolution landscape of *MULE* transposon in Arthropods

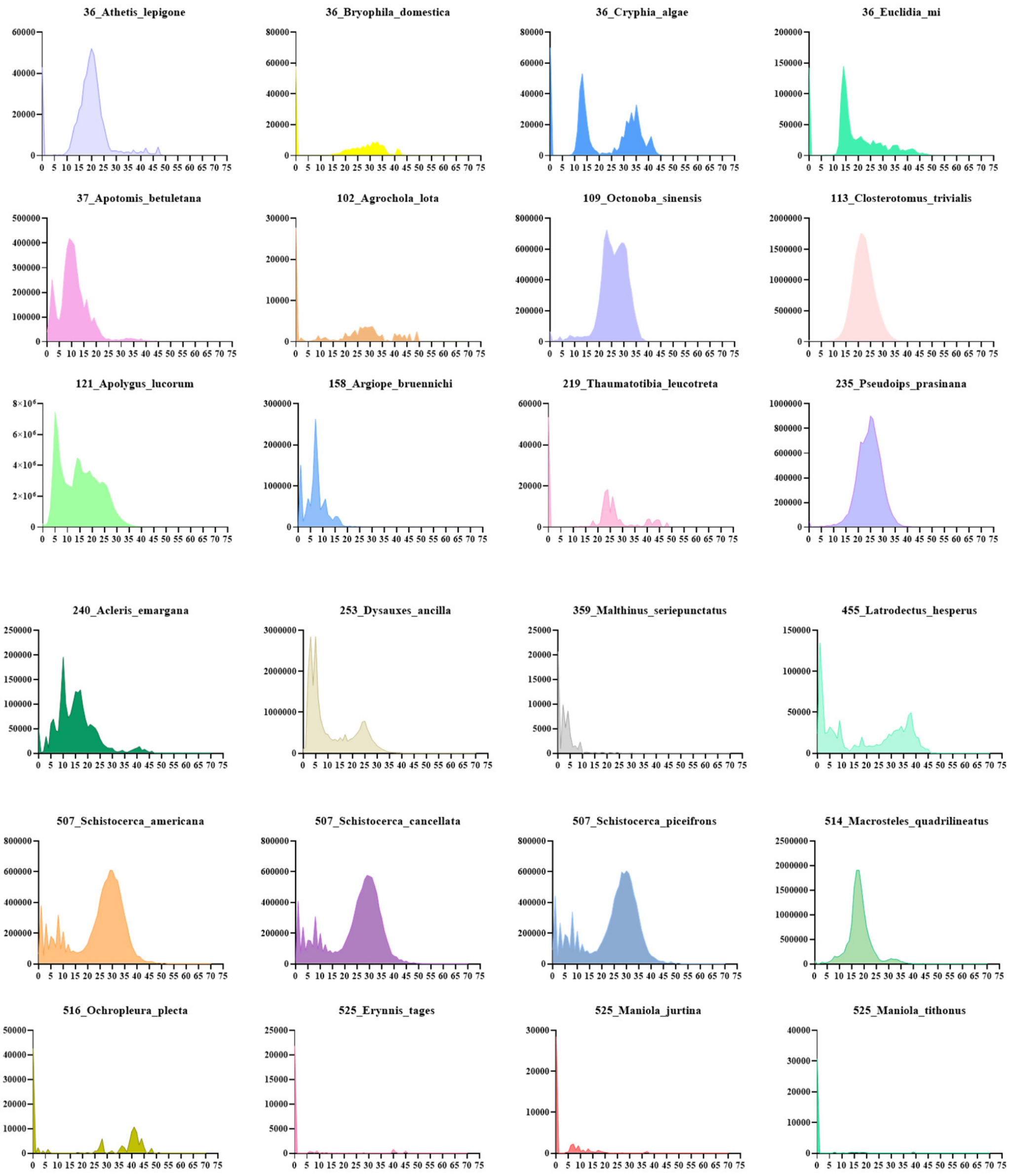

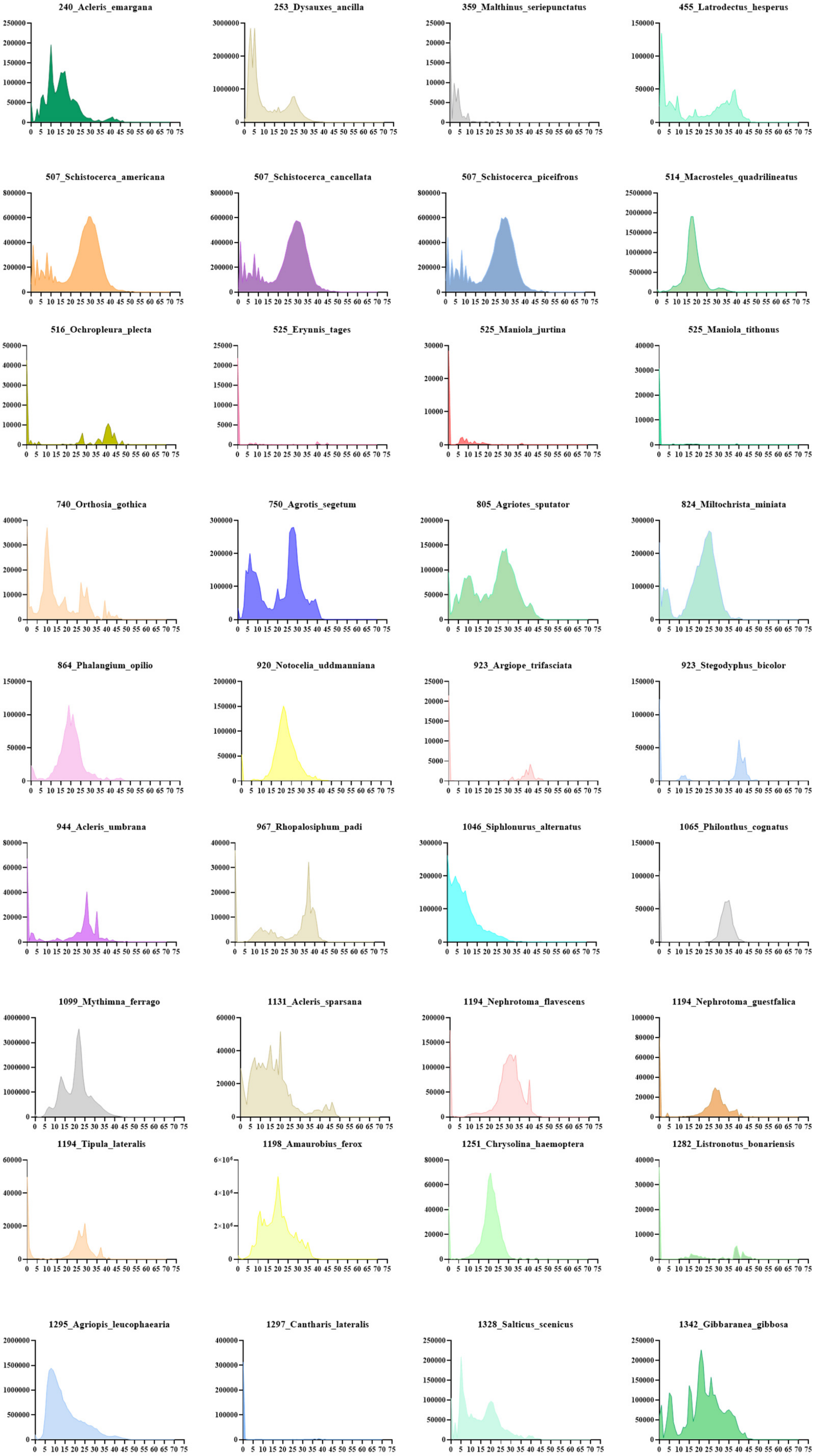
240_Acleris_emargana
253_Dysauxes_ancilla
359_Malthinus_seriepunctatus
455_Latrodectus_hesperus
507_Schistocerca_americana
507_Schistocerca_cancellata
507_Schistocerca_piceifrons
514_Macrosteles_quadrilineatus
516_Ochropleura_plecta
525_Erynnis_tages
525_Maniola_jurtina
525_Maniola_tithonus
740_Orthosia_gothica
750_Agrotis_segetum
805_Agriotes_sputator
824_Miltochrista_miniata
864_Phalangium_opilio
920_Notocelia_uddmanniana
923_Argiope_trifasciata
923_Stegodyphus_bicolor
944_Acleris_umbrana
967_Rhopalosiphum_padi
1046_Siphlonurus_alternatus
1065_Philonthus_cognatus
1099_Mythimna_ferrago
1131_Acleris_sparsana
1194_Nephrotoma_flavescens
1194_Nephrotoma_guestfalica
1194_Tipula_lateralis
1198_Amaurobius_ferox
1251_Chrysolina_haemoptera
1282_Listronotus_bonariensis
1295_Agriopis_leucophaearia
1297_Cantharis_lateralis
1328_Salticus_scenicus
1342_Gibbaranea_gibbosa

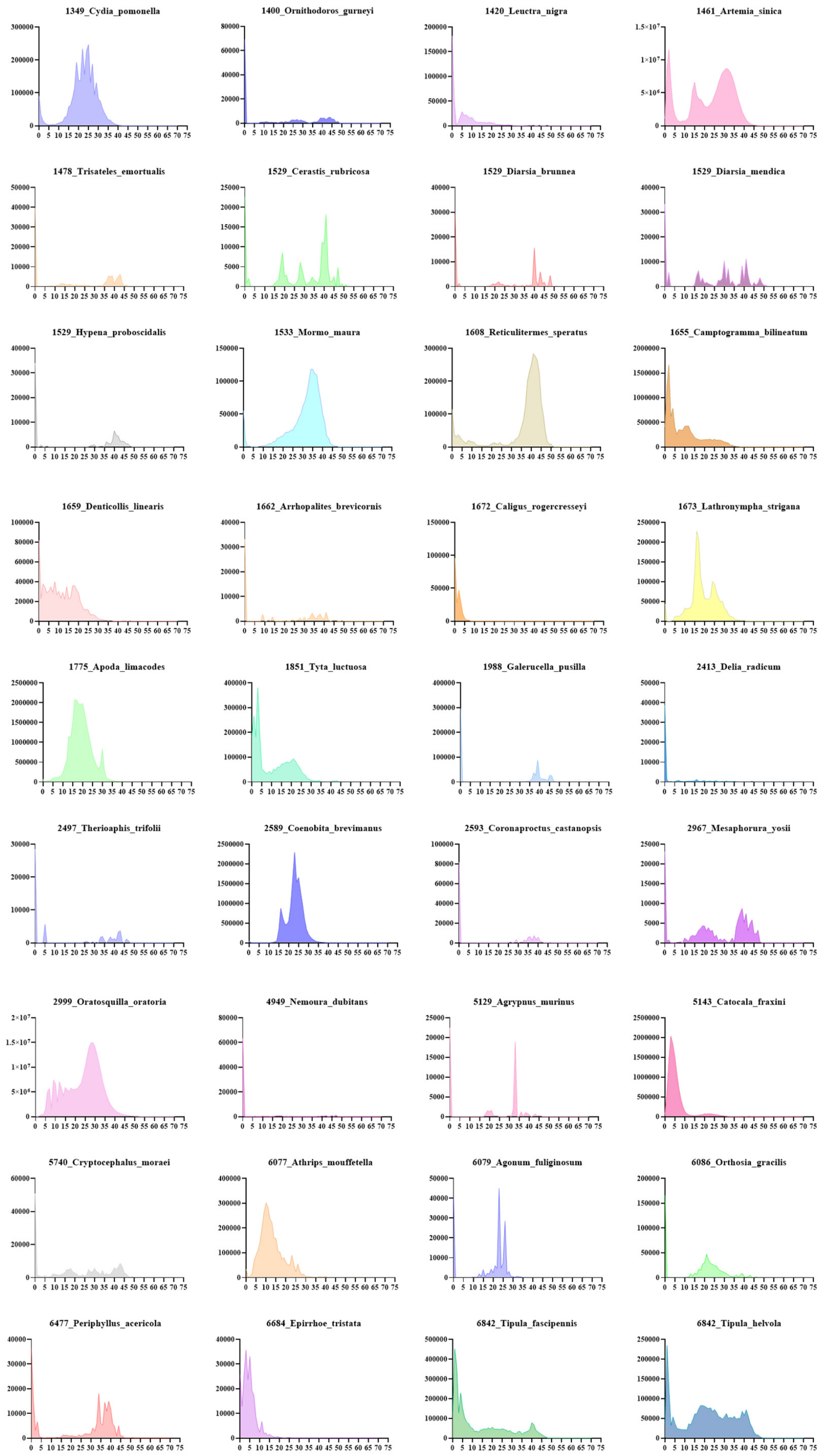
1349_Cydia_pomonella
1400_Ornithodoros_gurneyi
1420_Leuctra_nigra
1461_Artemia_sinica
1478_Trisateles_emortualis
1529_Cerastis_rubricosa
1529_Diarsia_brunnea
1529_Diarsia_mendica
1529_Hypena_proboscidalis
1533_Mormo_maura
1608_Reticulitermes_speratus
1655_Camptogramma_bilineatum
1659_Denticollis_linearis
1662_Arrhopalites_brevicornis
1672_Caligus_rogercresseyi
1673_Lathronympha_strigana
1775_Apoda_limacodes
1851_Tyta_luctuosa
1988_Galerucella_pusilla
2413_Delia_radicum
2497_Therioaphis_trifolii
2589_Coenobita_brevimanus
2593_Coronaproctus_castanopsis
2967_Mesaphorura_yosii
2999_Oratosquilla_oratoria
4949_Nemoura_dubitans
5129_Agrypnus_murinus
5143_Catocala_fraxini
5740_Cryptocephalus_moraei
6077_Athrips_mouffetella
6079_Agonum_fuliginosum
6086_Orthosia_gracilis
6477_Periphyllus_acericola
6684_Epirrhoe_tristata
6842_Tipula_fascipennis
6842_Tipula_helvola

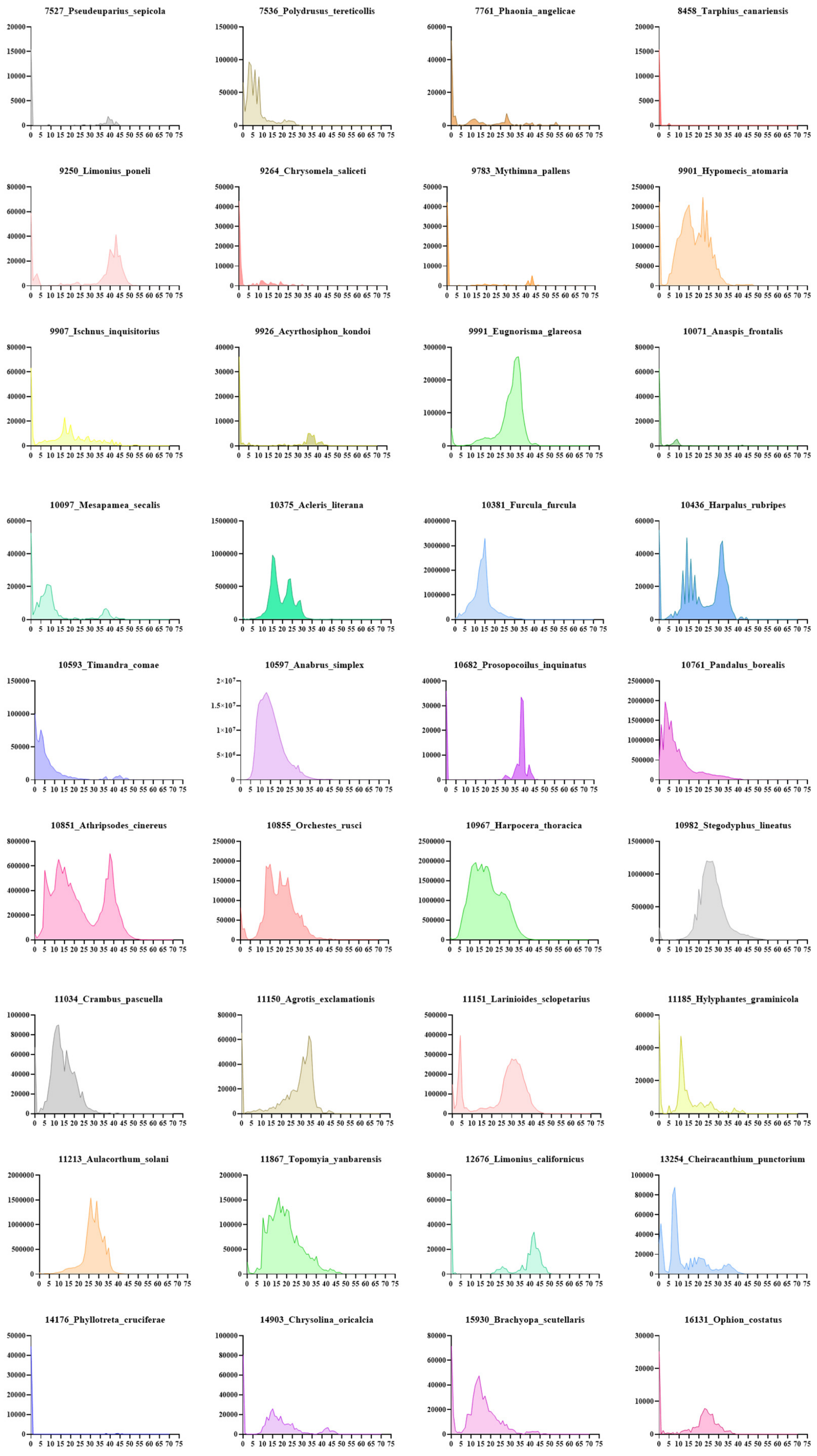
7527_Pseudeuparius_sepicola
7536_Polydrusus_tereticollis
7761_Phaonia_angelicae
8458_Tarphius_canariensis
9250_Limonius_poneli
9264_Chrysomela_saliceti
9783_Mythimna_pallens
9901_Hypomecis_atomaria
9907_Ischnus_inquisitorius
9926_Acyrthosiphon_kondoi
9991_Eugnorisma_glareosa
10071_Anaspis_frontalis
10097_Mesapamea_secalis
10375_Acleris_literana
10381_Furcula_furcula
10436_Harpalus_rubripes
10593_Timandra_comae
10597_Anabrus_simplex
10682_Prosopocoilus_inquinatus
10761_Pandalus_borealis
10851_Athripsodes_cinereus
10855_Orchestes_rusci
10967_Harpocera_thoracica
10982_Stegodyphus_lineatus
11034_Crambus_pascuella
11150_Agrotis_exclamationis
11151_Larinioides_sclopetarius
11185_Hylyphantes_graminicola
11213_Aulacorthum_solani
11867_Topomyia_yanbarensis
12676_Limonius_californicus
13254_Cheiracanthium_punctorium
14176_Phyllotreta_cruciferae
14903_Chrysolina_oricalcia
15930_Brachyopa_scutellaris
16131_Ophion_costatus

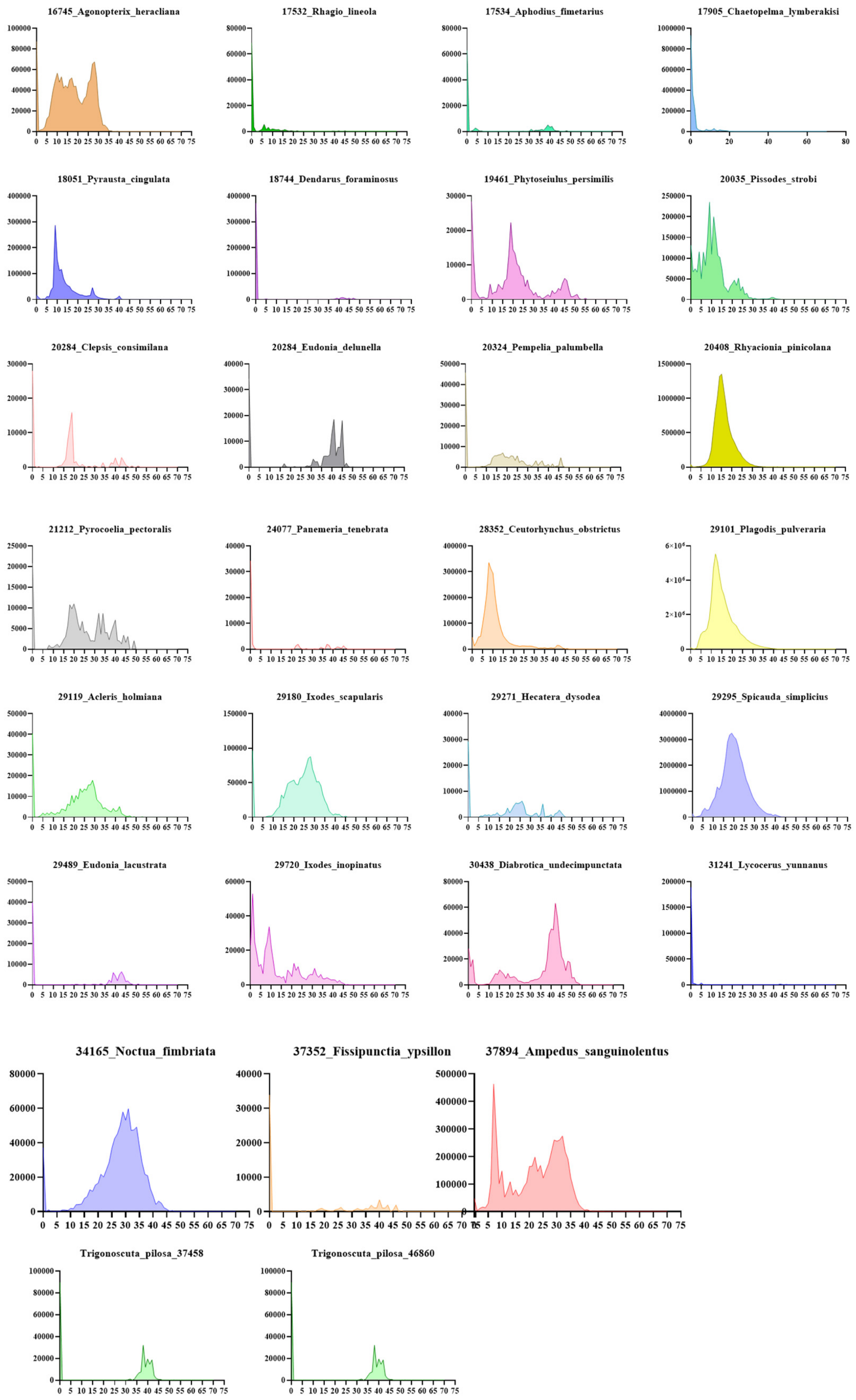
16745_Agonopterix_heracliana
17532_Rhagio_lineola
17534_Aphodius_fimetarius
17905_Chaetopelma_lymberakisi
18051_Pyrausta_cingulata
18744_Dendarus_foraminosus
19461_Phytoseiulus_persimilis
20035_Pissodes_strobi
20284_Clepsis_consimilana
20284_Eudonia_delunella
20324_Pempelia_palumbella
20408_Rhyacionia_pinicolana
21212_Pyrocoelia_pectoralis
24077_Panemeria_tenebrata
28352_Ceutorhynchus_obstrictus
29101_Plagodis_pulveraria
29119_Acleris_holmiana
29180_Ixodes_scapularis
29271_Hecatera_dysodea
29295_Spicauda_simplicius
29489_Eudonia_lacustrata
29720_Ixodes_inopinatus
30438_Diabrotica_undecimpunctata
31241_Lycocerus_yunnanus
34165_Noctua_fimbriata
37352_Fissipunctia_ypsillon
37894_Ampedus_sanguinolentus
Trigonoscuta_pilosa_37458
Trigonoscuta_pilosa_46860

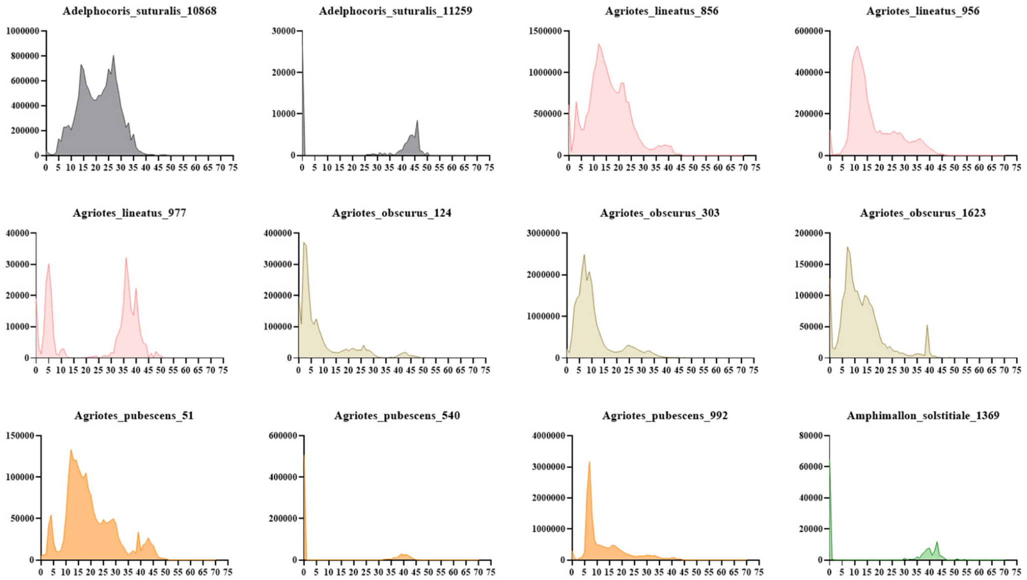
Adelphocoris_suturalis_10868
Adelphocoris_suturalis_11259
Agriotes_lineatus_856
Agriotes_lineatus_956
Agriotes_lineatus_977
Agriotes_obscurus_124
Agriotes_obscurus_303
Agriotes_obscurus_1623
Agriotes_pubescens_51
Agriotes_pubescens_540
Agriotes_pubescens_992
Amphimallon_solstitiale_1369

7.8 cm

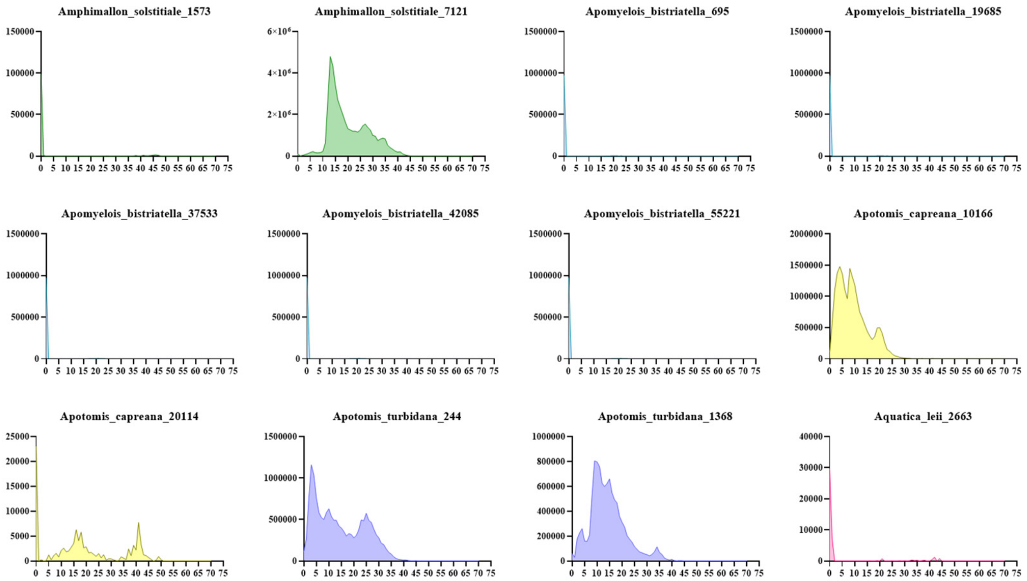
Amphimallon_solstitiale_1573
Amphimallon_solstitiale_7121
Apomyelois_bistriatella_695
Apomyelois_bistriatella_19685
Apomyelois_bistriatella_37533
Apomyelois_bistriatella_42085
Apomyelois_bistriatella_55221
Apotomis_capreana_10166
Apotomis_capreana_20114
Apotomis_turbidana_244
Apotomis_turbidana_1368
Aquatica_leii_2663

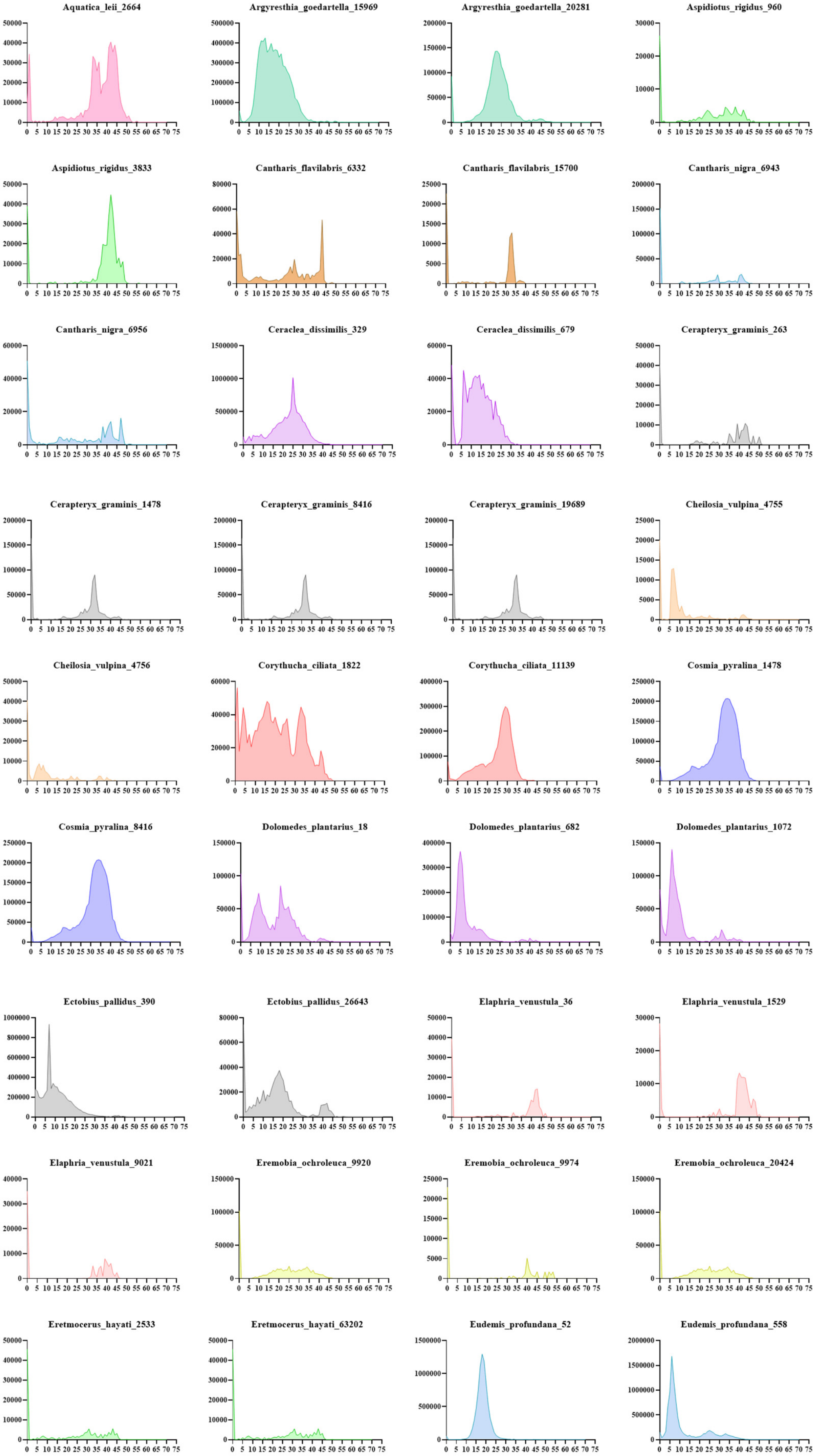
Aquatica_leii_2664
Argyresthia_goedartella_15969
Argyresthia_goedartella_20281
Aspidiotus_rigidus_960
Aspidiotus_rigidus_3833
Cantharis_flavilabris_6332
Cantharis_flavilabris_15700
Cantharis_nigra_6943
Cantharis_nigra_6956
Ceraclea_dissimilis_329
Ceraclea_dissimilis_679
Cerapteryx_graminis_263
Cerapteryx_graminis_1478
Cerapteryx_graminis_8416
Cerapteryx_graminis_19689
Cheilosia_vulpina_4755
Cheilosia_vulpina_4756
Corythucha_ciliata_1822
Corythucha_ciliata_11139
Cosmia_pyralina_1478
Cosmia_pyralina_8416
Dolomedes_plantarius_18
Dolomedes_plantarius_682
Dolomedes_plantarius_1072
Ectobius_pallidus_390
Ectobius_pallidus_26643
Elaphria_venustula_36
Elaphria_venustula_1529
Elaphria_venustula_9021
Eremobia_ochroleuca_9920
Eremobia_ochroleuca_9974
Eremobia_ochroleuca_20424
Eretmocerus_hayati_2533
Eretmocerus_hayati_63202
Eudemis_profundana_52
Eudemis_profundana_558

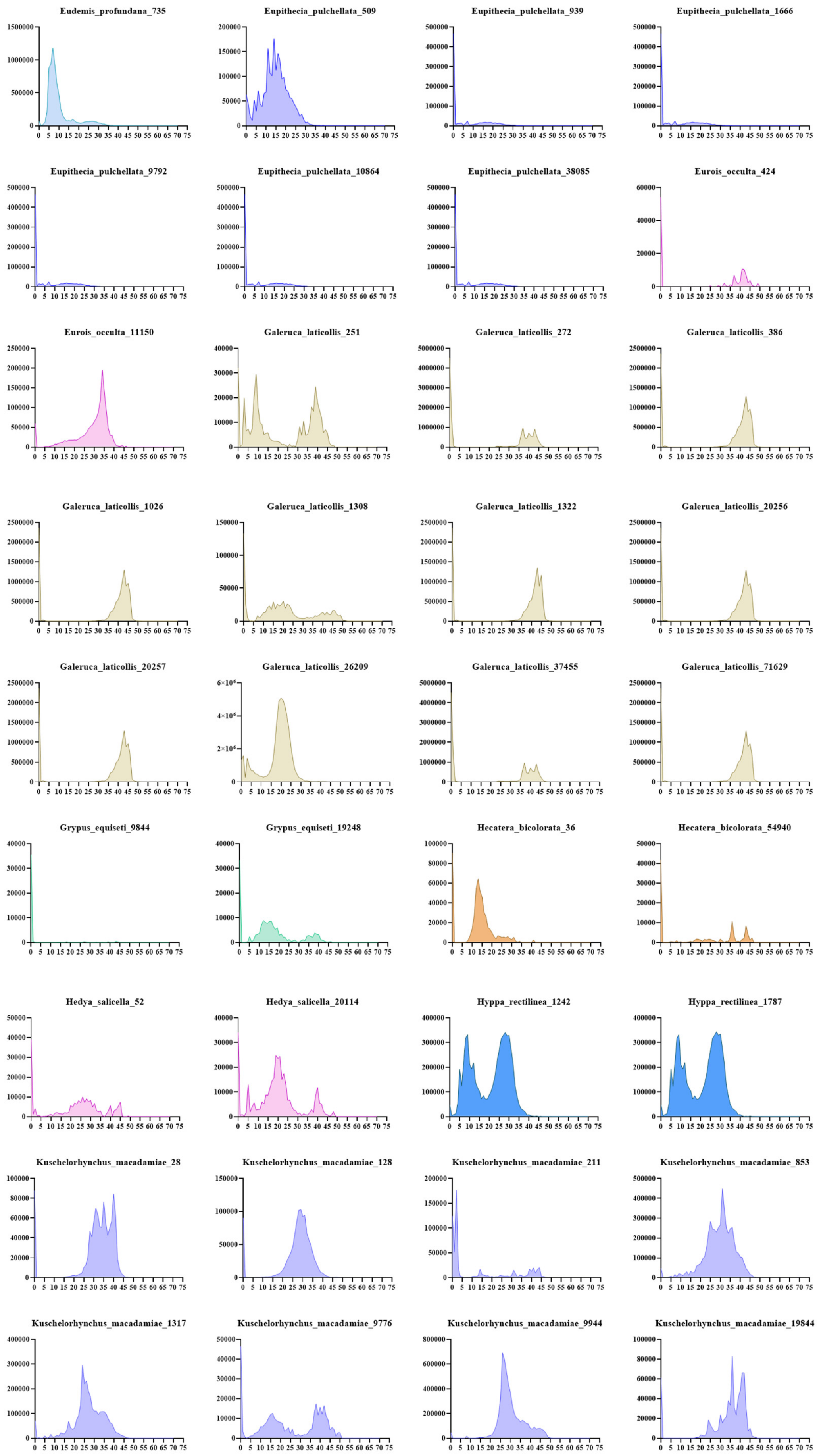
Eudemis_profundana_735
Eupithecia_pulchellata_509
Eupithecia_pulchellata_939
Eupithecia_pulchellata_1666
Eupithecia_pulchellata_9792
Eupithecia_pulchellata_10864
Eupithecia_pulchellata_38085
Eurois_occulta_424
Eurois_occulta_11150
Galeruca_laticollis_251
Galeruca_laticollis_272
Galeruca_laticollis_386
Galeruca_laticollis_1026
Galeruca_laticollis_1308
Galeruca_laticollis_1322
Galeruca_laticollis_20256
Galeruca_laticollis_20257
Galeruca_laticollis_26209
Galeruca_laticollis_37455
Galeruca_laticollis_71629
Grypus_equiseti_9844
Grypus_equiseti_19248
Hecatera_bicolorata_36
Hecatera_bicolorata_54940
Hedya_salicella_52
Hedya_salicella_20114
Hyppa_rectilinea_1242
Hyppa_rectilinea_1787
Kuschelorhynchus_macadamiae_28
Kuschelorhynchus_macadamiae_128
Kuschelorhynchus_macadamiae_211
Kuschelorhynchus_macadamiae_853
Kuschelorhynchus_macadamiae_1317
Kuschelorhynchus_macadamiae_9776
Kuschelorhynchus_macadamiae_9944
Kuschelorhynchus_macadamiae_19844

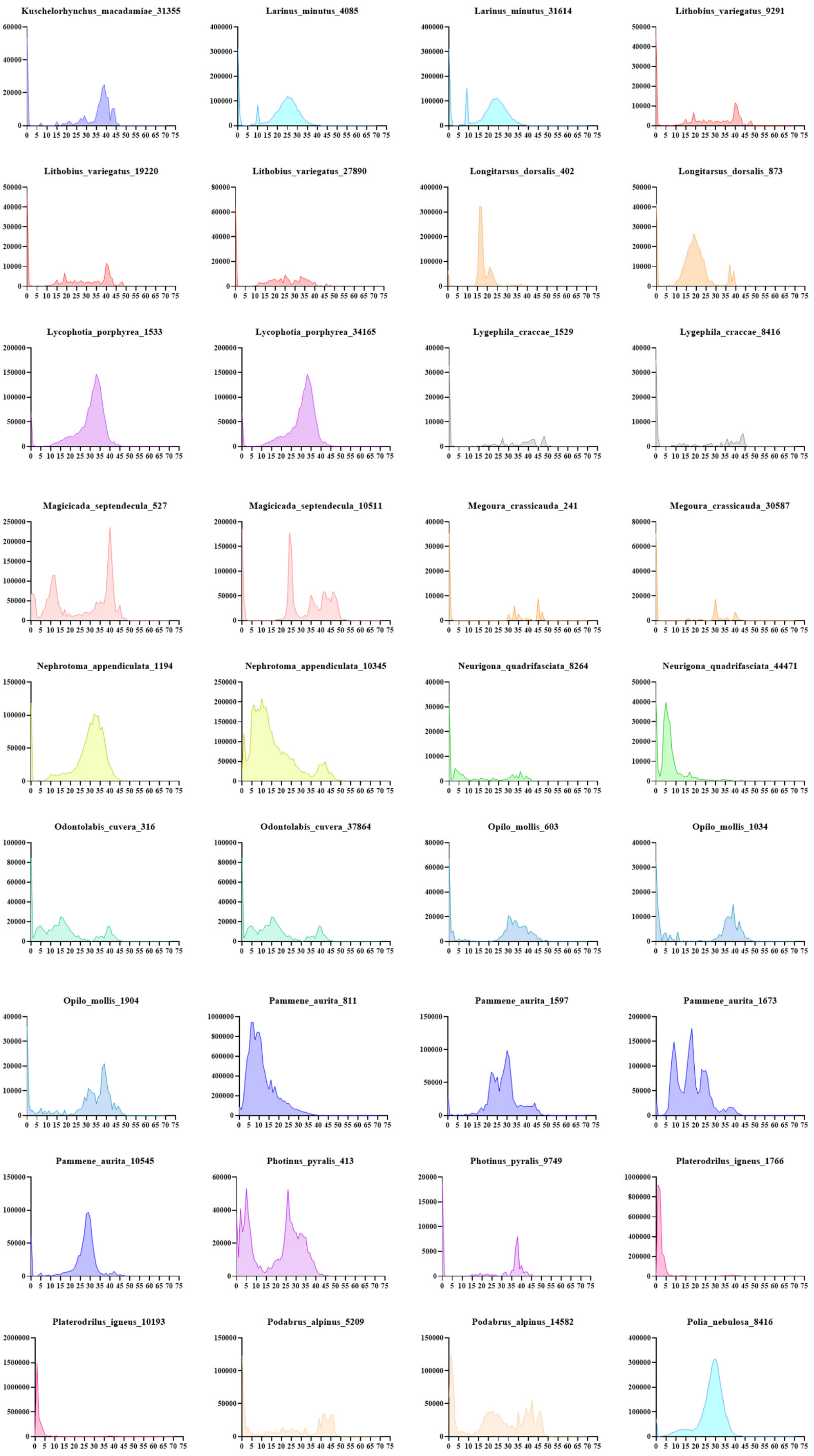

Kuschelorhynchus_macadamiae_31355
Larinus_minutus_4085
Larinus_minutus_31614
Lithobius_variegatus_9291
Lithobius_variegatus_19220
Lithobius_variegatus_27890
Longitarsus_dorsalis_402
Longitarsus_dorsalis_873
Lycophotia_porphyrea_1533
Lycophotia_porphyrea_34165
Lygephila_craccae_1529
Lygephila_craccae_8416
Magicicada_septendecula_527
Magicicada_septendecula_10511
Megoura_crassicauda_241
Megoura_crassicauda_30587
Nephrotoma_appendiculata_1194
Nephrotoma_appendiculata_10345
Neurigona_quadrifasciata_8264
Neurigona_quadrifasciata_44471
Odontolabis_cuvera_316
Odontolabis_cuvera_37864
Opilo_mollis_603
Opilo_mollis_1034
Opilo_mollis_1904
Pammene_aurita_811
Pammene_aurita_1597
Pammene_aurita_1673
Pammene_aurita_10545
Photinus_pyralis_413
Photinus_pyralis_9749
Platerodrilus_igneus_1766
Platerodrilus_igneus_10193
Podabrus_alpinus_5209
Podabrus_alpinus_14582
Polia_nebulosa_8416

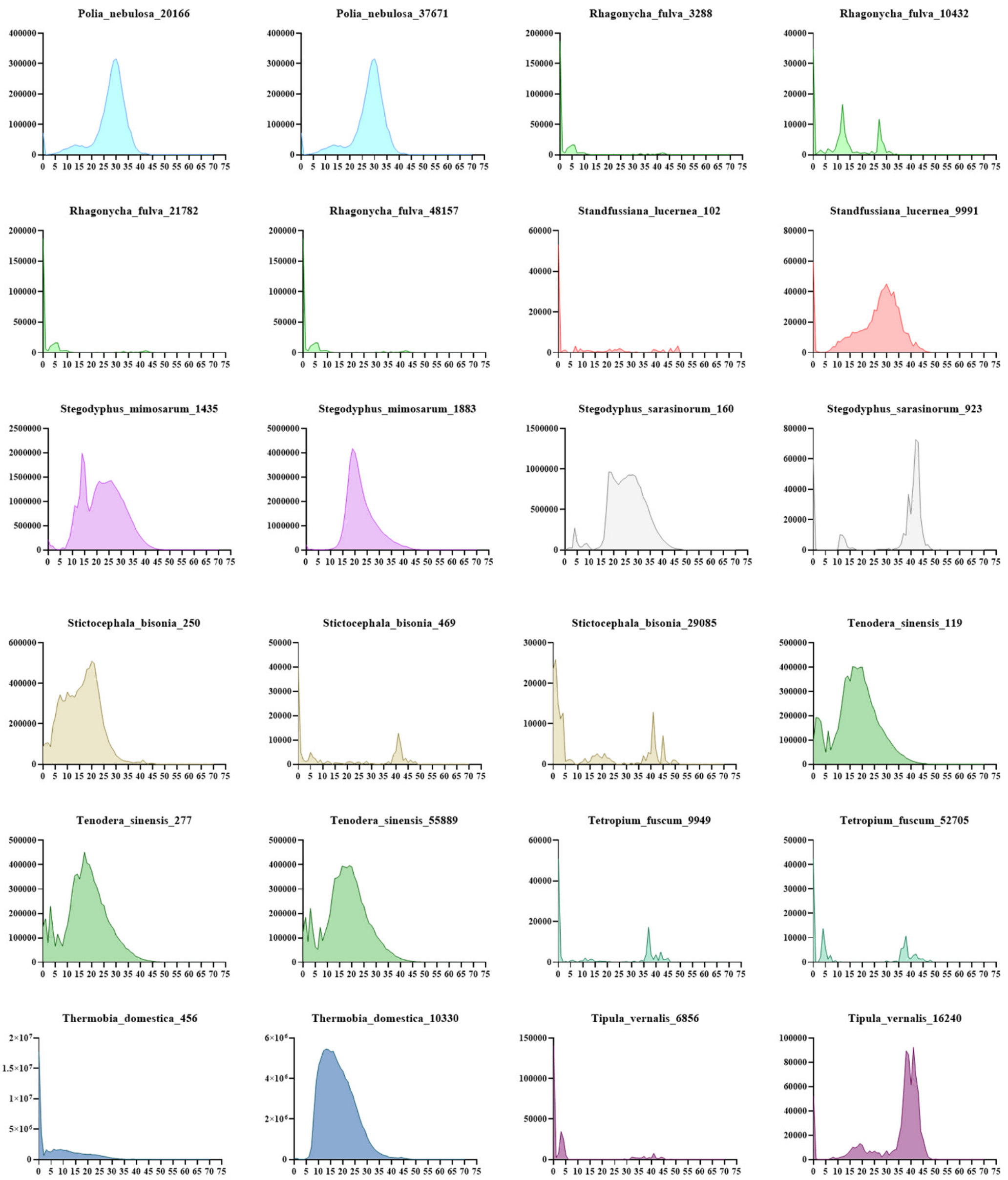

Figure S1: Evolutionary dynamics of *MULE* transposon in Arthropods. The transposon insertion ages were estimated based on K divergences as described in methods. Temporal dynamics are visualized as K divergence (X-axis) versus genomic coverage (Y-axis). Different species are represented by distinct colors. Transposons more likely to be active are highlighted in red. (As a supplement to the main text (Figure 3), all transposons shown have a copy number greater than 5. (For details regarding the sequences associated with the figure, please refer to Supplementary Table S1).

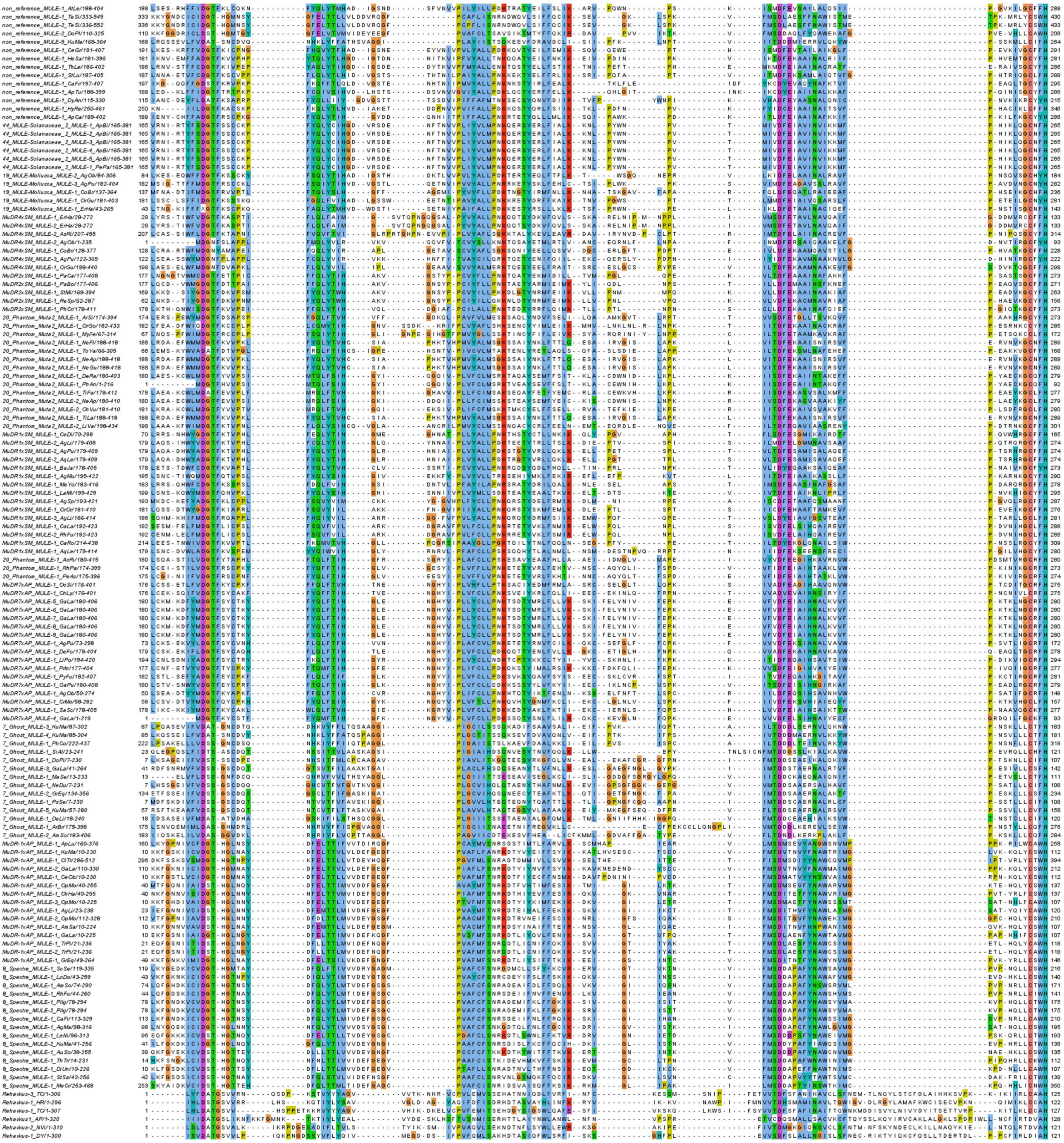

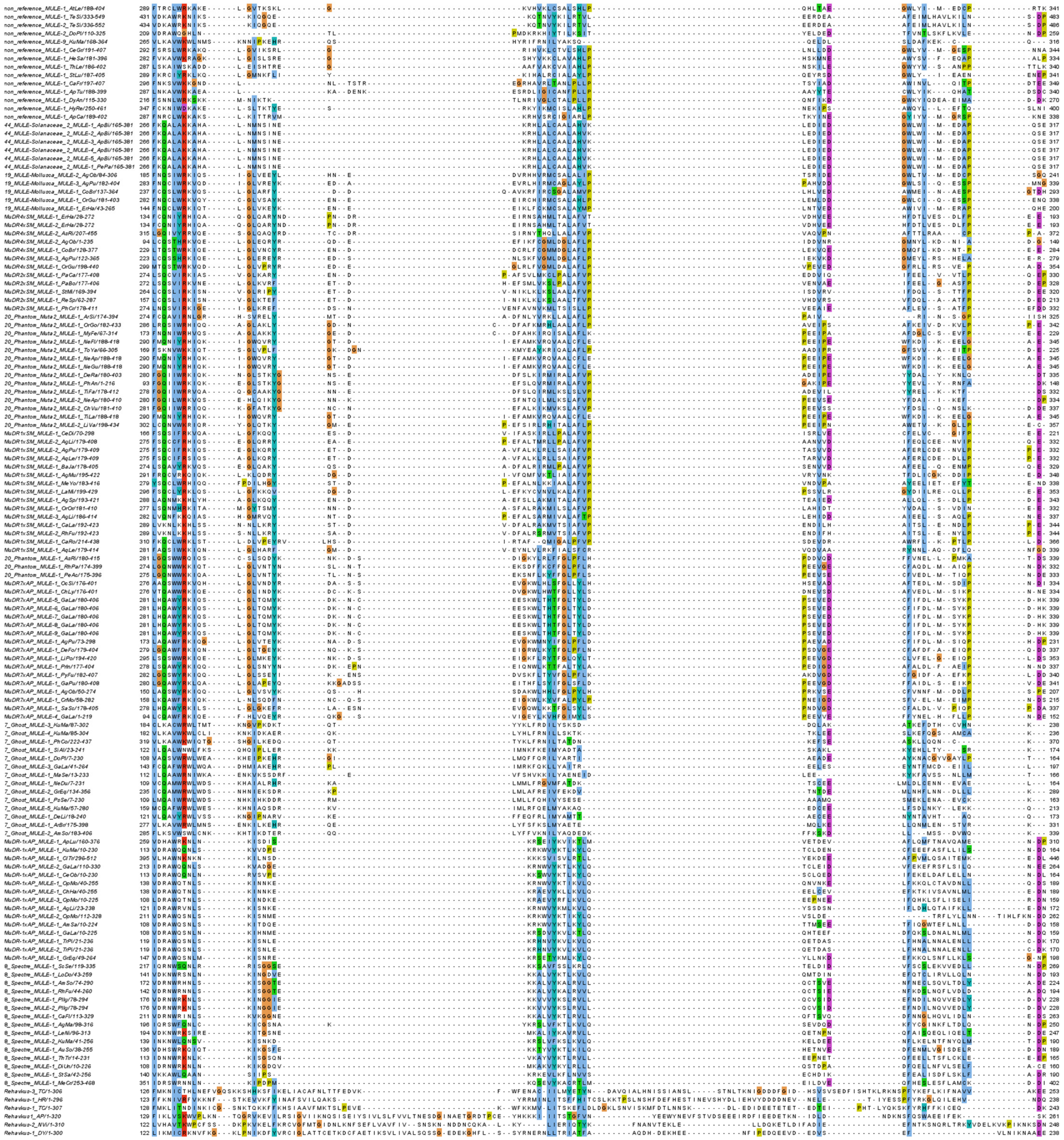

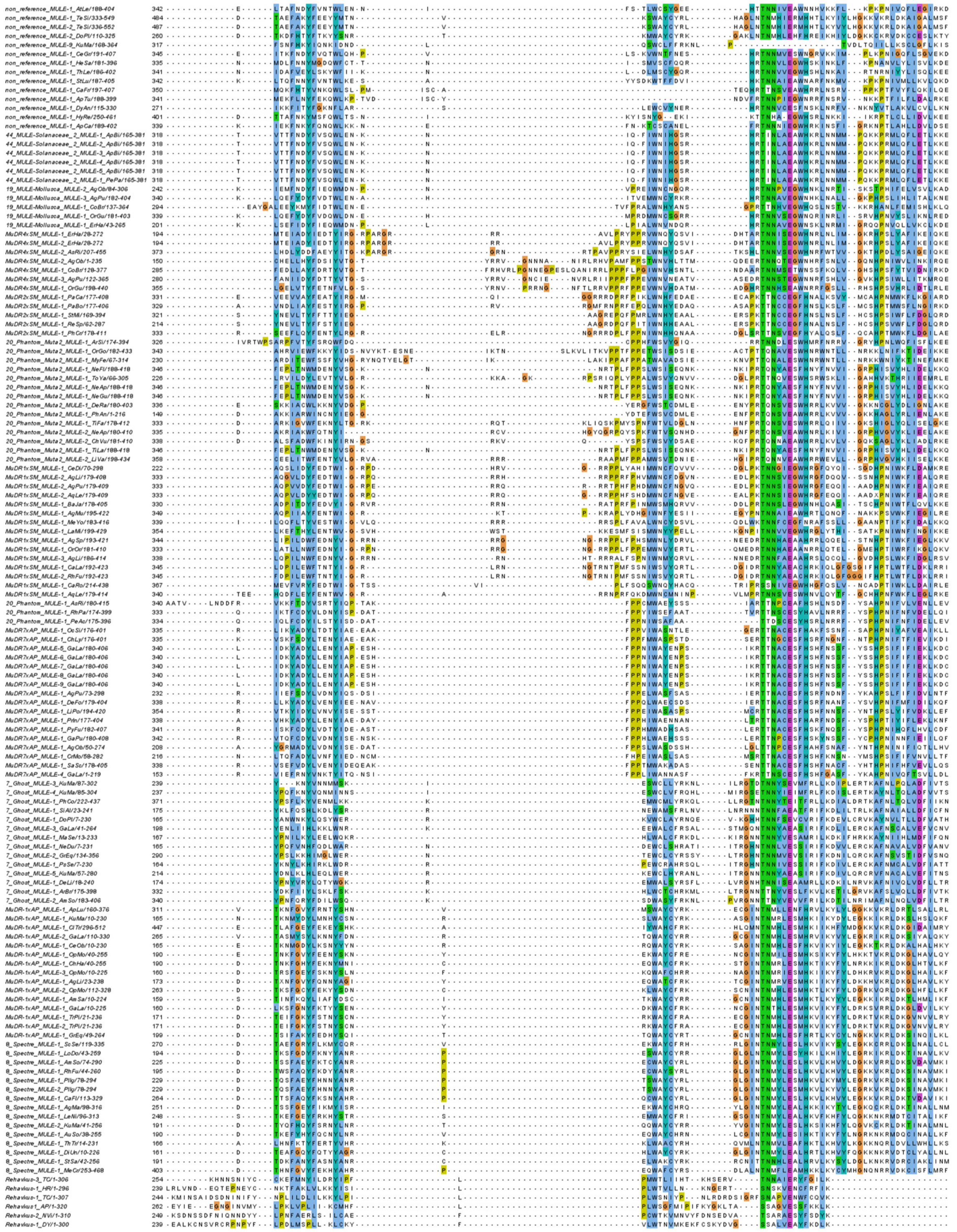

DDE

Figure S2: Alignment of amino acid sequences of partial *MULE* transposases from different clades in Arthropods. The catalytic domain (DDE) is indicated. Representative sequences from each clade are shown (randomly selected). See Supplementary Table S1 for the complete set of sequences.
